# The Experiences of Service Providers Who Care for Homeless Pregnant or Early Parenting Women

**DOI:** 10.1177/08445621251383062

**Published:** 2025-10-28

**Authors:** Kimberly Jarvis, Solina Richter, Vera Caine

**Affiliations:** 1 Faculty of Nursing, 7512 Memorial University of Newfoundland, St. John's, Newfoundland, Canada; 2 7235 College of Nursing, University of Saskatchewan, Saskatoon, Canada; 3 8205 School of Nursing, University of Victoria, Victoria, British Columbia, Canada

**Keywords:** feminist pragmatism, homelessness, pregnancy, parenting, structural vulnerability, women's health, trauma-informed care

## Abstract

**Background:**

Homelessness among women and children is a growing concern, shaped by intersecting structural inequities. Service providers working with women who are homeless, particularly those who are pregnant and/or parenting young children, navigate complex responsibilities that span legal, medical, housing, child welfare, and psychosocial domains. These responsibilities are often carried out in under-resourced environments and in response to trauma rooted in systemic injustice. A feminist pragmatist perspective recognizes the relational, embodied, and context-specific nature of this work, and values the insights of direct care providers as essential to shaping equitable and responsive care.

**Purpose:**

Our study purpose is to deepen our understanding of the ethical tensions and emotional and embodied labor inherent in the work of service providers who work with pregnant and/or parenting women who are homeless, while advocating for structural reforms that support both client outcomes and provider well-being

**Methods:**

This study is part of a larger community-based research initiative. This article draws on a subset of data from 22 semi-structured interviews and two focus groups with service providers, including social workers, nurses, nurse practitioners, psychologists, corrections staff, outreach workers, and health administrators. Data were analyzed thematically, guided by feminist pragmatist principles that center experience, reflexivity, and practical action.

**Results:**

Findings reveal persistent systemic barriers to care, including inadequate housing, fragmented services, and institutionalized discrimination. Despite these challenges, service providers expressed a deep sense of purpose and fulfillment in their work, rooted in relational engagement, advocacy, and bearing witness to the resilience of the women they support.

**Conclusion:**

Awareness of the human condition and a commitment to relational, justice-oriented care are central to effective service delivery. Health systems must prioritize equity and justice, ensuring that nurses and service providers are empowered and supported as advocates for pregnant and/or parenting women who are homeless.

Homelessness is a global crisis that transcends borders. While cross-country comparisons are challenging due to variations in definitions and data collection methods, the urgency of the issue is universal. This urgency has been intensified by a convergence of global challenges, including the lingering effects of the COVID-19 pandemic, a deepening housing affordability crisis, economic instability, forced migration, armed conflict, and the escalating impacts of climate change. These are compounded by systemic failures to adequately address substance misuse, mental health, education, child development, and gender equity, all of which are critical to long-term housing stability (Krahn et al., 2018). Notably, the crisis reflects a global shortfall in achieving the United Nations’ (2015) Sustainable Development Goals (SDGs), particularly those related to poverty (SDG 1), health and well-being (SDG 3), gender equality (SDG 5), and sustainable cities and communities (SDG 11). As of 2025, an estimated “2.8 billion people worldwide lack access to adequate housing, secure land, and basic water and sanitation services” (para.1), with over 300 million experiencing absolute homelessness (Chakma, 2025). The World Population Review (2025) highlights staggering rates of homelessness across the globe, including in the United States, often viewed as a global leader, Nigeria, the most populous country in Africa, and India, one of the two most populated nations in the world.

In Canada, homelessness remains difficult to quantify, particularly due to the prevalence of hidden homelessness, including residing in precarious housing. In 2023, it was estimated that between 150,000 and 300,000 people experienced homelessness across the country (Chan, 2024). The *National Shelter Study 2023 Update* reported that over 118,000 individuals accessed emergency shelters, with a growing proportion being women and children, especially in family shelters (Government of Canada, 2023). The *Government of Canada's Backgrounder: Action Research on Chronic Homelessness* (Government of Canada, 2024) emphasized, however, that homelessness in Canada is a pervasive and complex issue that extends far beyond what traditional shelter data can capture.

Women and children represent the fastest growing populations and are some of the most vulnerable populations affected by homelessness (UNAMIMA International, 2025). Their pathways into homelessness are often shaped by intersecting factors such as gender-based violence, poverty, and caregiving responsibilities (Women's National Housing & Homelessness Network, 2025). Recognizing these challenges, global leaders at the World Health Assembly (2025) reaffirmed the importance of prioritizing the health and well-being of women and children within the international development agenda. This commitment includes investing in community-based care, strengthening the health workforce, and ensuring equitable access to sexual and reproductive health services.

Supporting pregnant and parenting women who are homeless or precariously housed places multidimensional demands on frontline service providers. These women often face a combination of trauma, mental health challenges, substance use, domestic violence, and high-risk pregnancies, all of which require specialized, trauma-informed, and culturally sensitive care (Homeless Prenatal Program, 2024; Lakhanpaul & Svirydzenka, 2024). Service providers must often coordinate across fragmented systems, including health, housing, child welfare, and legal services, while managing high caseloads and limited resources. In the past, service providers have often focused on the logistical challenges women experienced (Schmidt et al., 2023). The emotional labor involved in supporting clients with histories of adversity and systemic marginalization can lead to burnout and compassion fatigue among service providers. Despite these challenges, many service providers (nurses, social workers, outreach workers, and mental health professionals) describe their work as deeply meaningful. Those who bear witness to the experiences of women in precarious housing situations often report a profound sense of purpose, personal growth, and professional fulfillment (UNAMIMA International, 2025).

In this study, we explored the complex interplay between structural vulnerability, institutional challenges, and the experiences of service providers working with pregnant and/or parenting women who experience homelessness. We trace how systemic underfunding and professional undervaluation not only constrain the quality and consistency of care but also contribute to a service provider's moral distress. At the same time, we emphasize the transformative power of authentic, compassionate relationships that challenge stigma, affirm a shared humanity, and offer moments of profound meaning and connection. We draw on feminist pragmatist ideas of social ethics and relational inquiry, emphasizing the importance of lived experience and social context (Addams, 1902). Key to this approach is the social and political engagement to find practical solutions that are informed by social justice (Hartrick Doane & Varcoe, 2021). Few studies have examined the experiences of service providers (Paisi et al., 2020). By centering on the experiences of service providers, this study aims to deepen our understanding of the ethical tensions and emotional and embodied labor inherent in this work, while advocating for structural reforms that support both client outcomes and provider well-being.

## Methodology and Methods

### Design and Setting

This study was conducted in Alberta, Canada, during a period of economic uncertainty, which contributed to elevated unemployment rates and increased demand for affordable housing. Since then, Edmonton has experienced a notable surge in individuals seeking shelter, driven by compounding factors such as the COVID-19 pandemic, ongoing economic pressures, and rapid population growth (Riebe, 2024). In response, the City of Edmonton (2024) has significantly expanded its financial commitment to addressing homelessness, increasing investments from $73.6 million in 2022 to over $91 million in 2023. These investments support a range of initiatives, including prevention strategies, emergency services, and long-term housing solutions.

In this paper, we present findings from a subset of participants (n = 22 service providers) drawn from a larger community-based research study focused on HIV prevention and care for women experiencing homelessness who are pregnant and/or parenting young children. Through close collaboration with these women, we engaged service providers involved in their care and conducted in-depth interviews. For this aspect of the study, we adopted a qualitative descriptive design. This design supported an in-depth examination of the complex and interdisciplinary dimensions of care provision, while also allowing for a nuanced understanding of the situational and historical contexts shaping service providers’ work. It illuminated how providers perceived themselves and navigated the challenges of their practice environments (Hartrick Doane & Varcoe, 2021). Furthermore, it fosters respect for the experiences of service providers while encouraging critical reflection on our positionality. Data were collected through individual face-to-face interviews and two focus groups to elicit insights into service providers’ experiences of providing care to women who are pregnant and/or parenting young children while experiencing homelessness.

Ethics approval was received through the Human Research Ethics Review Board at the University of Alberta (Pro00040818), and operational approval was obtained from Alberta Health Services. All participants provided written informed consent and were informed of the study's purpose, potential risks and benefits, and their rights, including privacy, confidentiality, and the option to withdraw from the study at any point without consequence.

### Recruitment and Sample

Participants were recruited using purposive sampling. Initial contact occurred through observation of service providers interacting with primary participants in the larger community-based research study. Service providers were invited to participate either via email or telephone contact. Each prospective participant received an information sheet outlining the study's purpose, procedures, and ethical considerations, and was asked to contact the research team if they were interested in participating.

The 22 service providers who participated represented both governmental and non-governmental community-based organizations. Inclusion criteria required that participants self-identify as working directly or indirectly with pregnant and/or early parenting women experiencing homelessness for at least one year. Participants represented a range of professional backgrounds, including social work, nursing, psychology, correctional services, community development, community outreach, and health administration. Of the 22 participants, 20 identified as women and two as men. Seven participants identified as Indigenous or as members of an ethnic minority group. Five participants disclosed having a personal history of homelessness, substance use, physical, sexual, or emotional abuse, and/or street involvement. Participants’ work experience ranged from 3 to 33 years, reflecting both early-career and seasoned professionals in the field.

### Data Collection

Service providers were interviewed at a time and location of their choosing. Nineteen participants opted to be interviewed at their place of employment. Individual face-to-face interviews served as the primary method of data collection, supplemented by two focus groups. The use of multiple methods was intended to strengthen the study's trustworthiness through triangulation, allowing for a more comprehensive understanding of the phenomenon under investigation. Semi-structured interview guides were used for both individual and focus group interviews. Questions focused on service providers’ experiences working with women who were pregnant and/or parenting young children and experiencing housing instability; the availability and nature of programs, supports, and services for this population; perceived or actual gaps in care; and strategies service providers used to sustain themselves in the provision of care.

Interviews ranged from 60 to 90 min in duration. All interviews were audio-recorded with participants’ consent, and detailed field notes were made following each session. Data collection continued until sufficient depth and richness were achieved, yielding comprehensive insights into the lived experiences and perspectives of the participating service providers.

### Data Analysis

Interviews were transcribed verbatim, and data analysis followed the iterative, non-linear process outlined by Roper and Shapira (2000), which includes coding, sorting, generalizing, and memoing. Transcripts were read line-by-line, with significant words, phrases, and passages highlighted and assigned codes based on their relevance to the research question. These initial codes were then sorted into patterns to identify emerging themes and broader conceptual categories. The process of generalizing allowed for the identification of relationships and linkages across the data, facilitating the development of a conceptual framework. Reflexive memos were recorded throughout the analysis to deepen interpretive insights and inform implications for practice.

Trustworthiness was established through several strategies. The involvement of multiple researchers in data collection helped minimize interviewer bias; researchers engaged in continuous conversations to ensure an iterative review of the interview guide and that new learnings informed further conversations. Peer debriefing contributed to the accuracy of interpretations and the resolution of contradictory findings. Member checking was also employed; research team members included long-term service providers. For example, participants were invited to engage in the preliminary interpretations, offering opportunities to co-construct and validate the findings. Given that many of the participants identified as Indigenous, support from an Indigenous Elder was also sought to ensure the cultural integrity and relevance of the research process.

## Findings

The findings of this study illuminate the intricate realities faced by service providers supporting pregnant and parenting women who are homeless. Through their narratives, key themes emerged that highlight the interplay of structural vulnerability, institutional challenges, and meaning inherent in their work. The themes provide a nuanced understanding of the challenges and motivations shaping service provision within this complex context.

### Structural Vulnerability

Service providers in this study acknowledged the profound impact of structural vulnerability on their experiences of providing care, as well as the experiences they witnessed in the women they worked with. Structural vulnerabilities recognize that social structures both create and reinforce vulnerabilities. Linking this to feminist pragmatism means to look closely at the experiences and to highlight ways in which oppressive structures are challenged through practical action and social change. Service providers recognized that the traumatic life circumstances experienced by women and children they serve, marked by homelessness, poverty, and systemic marginalization, create significant barriers to meaningful change.

You hear these stories of all these things these women go through on a daily basis to survive. You spend weeks building them up to go to the hospital, go to these appointments, and then [mainstream] service providers are like ‘you’re fucking using drugs, I don’t wanna help you’

These challenges are compounded by mainstream service systems that often remain unsupportive.

‘She lost her place, so go back to the shelter.’ [Now] we start back at square one. But there's no shelters available for women and their children. I think … five beds or seven beds. How many pregnant women and families struggle with homelessness in this city? A lot more than five or seven.

Even more troubling for some service providers was the disillusionment they experienced when colleagues or others within their profession expressed judgmental or discriminatory attitudes toward the women they served. This lack of recognition for ethical and compassionate professional practice was particularly disheartening, as one nurse explained:

One of the biggest ethical issues I always see is just people being treated like shit. It's like we’re [professionals] taught these standards of care, and we’re taught our ethics in school … and they’re not following them, they’re not treating people with respect. They’re acting like the moral police, which isn’t what we’re there to do.

Such environments undermine clients’ dignity and reflect broader social inequities, which in turn affect the service providers, questioning their capacity to make a lasting difference.

Being exposed to, being touched by people's life stories, being put in a place where of deep empathy, where I would think if I had that sort of life, where would I be? Could I have made it, would I have made it? Those kinds of reflections, and then you know, carrying that as sort of a duty ‘cause with knowledge comes responsibility.

This sense of responsibility drives some service providers to consider how they respond to vulnerability matters. They articulate how they consistently challenge attitudes, recognizing that this is a fundamental aspect of the human condition (Gilson, 2013).

### Institutional Challenges

Service providers working with pregnant women who are homeless frequently encounter significant resource constraints that complicate their ability to deliver effective care; it also shapes ethical judgements. Feminist pragmatism makes visible that institutions can be sites of oppression as well as sites that can create social change (Stanford Encyclopedia of Philosophy, 2025). Underfunded and overstretched programs contribute to a pervasive sense of devaluation and powerlessness among frontline workers, limiting both the scope and quality of services. One nurse highlighted the disparity in compensation, noting that their salary at a community agency was approximately 30% lower than that of a similarly qualified colleague employed by the provincial government. Such inequities in remuneration foster psychological frustration and a sense of disempowerment. As one outreach worker poignantly observed:

A lot of frontline workers are one paycheque away from their clients. So, when you have all that stress in your own life and no access to resources or what you need for resources, then you come, you work in this hard-ass work every day, and you don’t have any support systems—and money is part of that.

In addition to financial strain, service inconsistency further undermines trust in the system. Many mainstream programs operate on rigid schedules, typically 9 to 5, Monday to Friday, which do not align with the unpredictable and urgent needs of the homeless population. Even services designed specifically for this demographic are often unreliable. A provider noted,

Another program right now that our women access … they’re not very consistent. They’re not open this day; they might be open that day. This lack of continuity leaves clients feeling unsupported and fosters skepticism toward the healthcare system and its providers.

Unfortunately, the policy-making process itself is often experienced as performative rather than participatory. Although service providers are frequently invited to consultations, many feel their input is disregarded. As one provider explained,

They [policy makers] consult. I’ve never been consulted so much in my whole life, but it is a disingenuous system because they have already decided … If you don’t participate, it's like, ‘Well, we gave people a chance and they didn’t participate.’ And if you do participate, it's like you’re putting the stamp on whatever crazy nonsense they come up with.

These systemic issues not only hinder client outcomes but also exacerbate the frustration and emotional burden experienced by those striving to support a highly marginalized population within an insufficiently resourced system. Despite the clear understanding of systems being oppressive, service providers continuously engaged with system challenges and saw this as a place for social change.

### Advocating for Justice

Service providers consistently highlighted the punitive and unsupportive nature of the social systems that women who are homeless and their children must navigate to access care. Rather than offering protection or support, these systems often exacerbate harm, particularly for women already experiencing structural vulnerability. As one service provider reflected,

If I could prevent any families from going through that system, especially with their experiences [of trauma] … you know, maybe we’d give their children a better chance.

This statement underscores a profound critique; that the very systems designed to support families are, in practice, contributing to their distress and instability.

In response to these systemic failures, many service providers described stepping into advocacy roles that extended well beyond their formal job descriptions. One community outreach worker recounted accompanying a mother to a bail hearing for a minor infraction, to ensure she would not be ‘run over’ by the system. The service provider explained:

She's scared because she thinks she’ll be arrested, and she's just terrified to go by herself, so I go with her. There's not a lot of gentleness or kindness in any of the systems, I think … it keeps re-injuring people.

This lack of compassion and trauma-informed care is particularly evident in the child welfare system, where women experiencing homelessness often face the threat of losing custody of their children. Service providers described the emotional devastation that follows child apprehension, noting that it frequently leads to increased substance use, deepened trauma, and diminished self-worth. One outreach worker shared a particularly harrowing example:

One time, a social worker came and presented an apprehension to one of my moms who hadn’t even delivered … hadn’t even delivered. Then I had to support the mother through delivery knowing that Child Welfare was right there to swoop and scoop.

Service providers also bore witness to the compounded discrimination faced by Indigenous women, who are disproportionately affected by homelessness and child apprehension (Alberton et al., 2020). These women often experience re-traumatization within systems that reflect and reinforce white privilege. One service provider noted the deep mistrust Indigenous women feel toward institutional services:

There is a lack of understanding and compassion for the situations these women find themselves in. Instead of providing resources and programs, a lot of the moms talk about … they’re like, the Indian Residential School never went away, they just call it Child Welfare now. A lot of the time, the nurses and social workers are white. How many Children's Services workers do you see that are Aboriginal? How many nurses do you see that are Aboriginal? So again, that's why they don’t want to talk.

These reflections reveal the emotional and ethical burden service providers carry as they navigate systems that often perpetuate harm. Their advocacy is not only a professional responsibility but a moral imperative, rooted in solidarity with the women they serve and a commitment to justice, equity, and systemic change.

### Compassion in Practice: Joy, Meaning, and the Emotional Realities

Reflective of feminist pragmatist ideas, despite the structural and resource-based challenges they face, service providers consistently emphasized a deep moral and ethical commitment to their work. Many described their roles not merely as work, but as professions grounded in compassion, justice, and a belief in the dignity of every client. As one provider reflected, “There's lots of success stories. To me, the most important success is when somebody moves to a better place in their life.” Importantly, these “better places” were not always defined by dramatic transformations. Rather, providers celebrated the small, incremental success that marked a woman's journey toward stability and self-determination. One service provider explained, “I changed my clothes today. That I came to my doctor's appointment … I called my worker. It's just minuscule steps. But you celebrate every inch of the way.” The tone in their voice conveyed genuine joy and pride in witnessing these moments. These everyday successes, though often invisible to the broader system, were deeply meaningful to direct care providers and served as powerful reminders of the value and impact of their work.

Service providers consistently emphasized the authenticity, respect, and mutual reward embedded in the relationships they developed with their clients. These connections were not only central to effective care but also deeply transformative for the providers themselves. Many described how their work prompted reflection on complex ethical questions and reshaped their own identities. As one provider expressed, “We’re better people for who they are and what they share.” This sentiment was echoed by others who found their most meaningful experiences rooted in a desire to understand and affirm the humanity of the women they served. One provider, whose personal history mirrored that of many clients, shared:

Everyone has a story. You have a story; I have a story. Listen to their stories and they’ll tell you … First of all, they’re a person. They’re someone's daughter, granddaughter, cousin, and niece. Crack is just what they do. First and foremost, they’re a person.

This capacity to see, hear, and acknowledge the person beyond their circumstances reflects the deep compassion and ethical commitment that service providers bring to their work. It also makes visible their close attention to experience. All providers in this study expressed a shared passion and sense of purpose, as captured in one provider's reflection:

I’m exactly where I’m supposed to be. I started working in this program with the ladies and with the staff and fell in love … I really do believe we are a global community, and the sooner we all get to the point where we are taking care of all our community's most vulnerable … [the sooner we’ll have] a healthier global community.

Despite this passion and commitment, providers acknowledged that the structural and institutional pressures surrounding their work often threatened the sustainability of both their programs and their well-being. The tension between the transformative nature of their relationships and the structural limitations of the system underscores the urgent need for support that honors both the humanity of clients and the dedication of those who serve them.

## Discussion

The relationship between service providers and the women they support is not merely therapeutic; it is a deeply human and ethical engagement grounded in the principles of holistic, person-centered care. Service providers’ philosophical approaches are strongly influenced by the lived experiences of their clients, which mirrors approaches in feminist pragmatism. As Addams (1902) pointed out, it is critical for those in leadership positions to “move with the people” (p. 69) and to ground their knowledge and care in the social context of the people they serve. Service providers in this study, particularly those who were nurses, working with women who are homeless and are pregnant or parenting young children, describe their practice as relational, empathetic, and rooted in mutual respect. This connection transcends clinical boundaries, reflecting a commitment to seeing each woman not as a diagnosis or a social condition, but as a person with inherent dignity and resilience. As Brown (2021) notes, such relationships are a source of sustenance and strength, embodying the essence of compassionate care.

Understanding the needs of this population requires service providers to move beyond biomedical models and embrace a social determinant of health framework. Homelessness, substance use, and mental health challenges are not isolated issues; they are interwoven with systemic inequities, trauma histories, and structural violence. When service providers, in this study, invited women to share their stories, they were engaging in a practice of ethical listening, which Cameron (2004) describes as a form of care that is intentional, accountable, and deeply relational. This approach aligns with nursing values of advocacy, equity, and justice. It positions the nurse not only as a caregiver, but as a witness to the human experience. Grounding themselves in the experiences of women also recognises that there are no universal solutions. It is Siegfried (1996), a feminist pragmatist scholar, who reminds us that being grounded in experiences helps us avoid imposing and prioritizing a fixed reality.

Working from a strengths-based perspective, service providers recognize and affirm the capacity of women to heal, grow, and reclaim agency. This orientation challenges deficit-based narratives and aligns with nursing's commitment to empowerment and health promotion. Stevens et al., (2024) found that focusing on strengths enhances professionals’ ability to recognize client capabilities and fosters collaborative, transformative care. The establishment of human connection builds improved health outcomes and overall well-being. Service providers described the profound emotional and professional fulfillment that comes from witnessing women develop confidence, autonomy, and self-determination. There is evidence to support that when service providers promote opportunity and choice, they experience increased engagement and reduced compassion fatigue, highlighting the reciprocal nature of relational care (Lenzi et al., 2021).

When working in marginalized settings, service providers often face systemic barriers that undermine their practice. Many in this study reported feeling excluded from institutional decision-making, undervalued in comparison to colleagues in more traditional roles, and stigmatized due to their association with populations experiencing homelessness. This reflects Goffman's (1963) concept of stigma by association, where the dehumanization of clients extends to those who care for them. Belcher and DeForge (2012) emphasize that individuals experiencing homelessness are frequently perceived as ‘less than fully human,’ and this perception can taint the professional identity of nurses who serve them. The notion of ‘dirty work’ (Douglas, 2001; Hughes, 1962) remains relevant, contributing to chronic underfunding and inadequate support for service providers in these roles.

In response to these challenges, service providers embrace advocacy as both a professional and moral imperative. Rooted in the *Code of Ethics for Nurses* (International Council of Nursing, 2021), advocacy involves challenging unjust systems, amplifying marginalized voices, and ensuring that care is equitable, transparent, and responsive, as well as reflective of the ordinary lives of people. Service providers in this study described advocacy not as an abstract ideal but as a lived practice, speaking out against punitive policies, resisting paternalism, and standing in solidarity with women navigating hostile environments. Advocacy is a shared commitment to justice, grounded in trust and mutual respect, and highlights the importance of care delivery (Purkey & MacKenzie, 2019).

By centering the experiences of service providers, this study offers insights for health system leaders, educators, and policymakers. It calls for a reimagining of care systems that honor both the complexity of clients’ lives and the expertise of service providers who support them. A strengths-based, relational approach is not only clinically effective, but also ethically necessary. It affirms the dignity of both client and caregiver, fostering a culture of care that promotes wellness, equity, and human flourishing.

## Conclusion

Nurses and frontline providers, working at the nexus of systemic barriers and client care, face the dual burden of chronic underfunding and the ethical and emotional labor inherent in this work. Yet, they also foster transformative, compassionate relationships that affirm dignity and resilience. Health systems must prioritize equity and justice, ensuring that nurses and service providers are supported as advocates for pregnant and/or parenting women who experience homelessness.

## References

[bibr1-08445621251383062] Addams J. (1902). Democracy and social ethics. The Macmillan Company.

[bibr2-08445621251383062] Alberton A. M. Angell G. B. Gorey K. M. Grenier S. (2020). Homelessness among indigenous peoples in Canada: The impacts of child welfare involvement and educational achievement. Children and Youth Services Review, 116, 105256. 10.1016/j.childyouth.2020.105256

[bibr3-08445621251383062] Belcher J. R. Deforge B. R. (2012). Social stigma and homelessness: The limits of social change. Journal of Human Behavior in the Social Environment, 22(8), 929–946. 10.1080/10911359.2012.707941

[bibr4-08445621251383062] Brown B. (2021). Atlas of the heart: Mapping meaning connection and the language of human experience. Penguin Random House.

[bibr5-08445621251383062] Cameron B. (2004). Ethical moments in practice: The nursing ‘how are you?’ revisited. Nursing Ethics, 11(1), 53–62. 10.1191/0969733004ne666oa 14763650

[bibr6-08445621251383062] Chakma . (2025, May 29). UN searches for solutions to global housing crisis. *UN News: Global perspective Human stories*. https://news.un.org/en/story/2025/05/1163851

[bibr7-08445621251383062] Chan J. (2024, January 28). Canada’s homelessness crisis: What’s gone wrong since the 1960s? *The Varsity*. https://thevarsity.ca/2024/01/28/canadas-homelessness-crisis-whats-gone-wrong-since-the-1960s/

[bibr8-08445621251383062] City of Edmonton . (2024). *Homelessness and housing services plan*. https://www.edmonton.ca/sites/default/files/public-files/assets/PDF/Homelessness-Housing-Services-Plan.pdf

[bibr9-08445621251383062] Douglas M. (2001). Purity and danger: An analysis of the concepts of pollution and taboo. Routledge.

[bibr10-08445621251383062] Gilson E. (2013). The ethics of vulnerability: A feminist analysis of social life and practice (1st ed.). Routledge. 10.4324/9780203078136

[bibr11-08445621251383062] Goffman E. (1963). Stigma: Notes on the management of spoiled identity. Prentice-Hall.

[bibr12-08445621251383062] Government of Canada . (2023). *Homelessness data snapshot: The National Shelter Study* 2023. https://housing-infrastructure.canada.ca/alt-format/pdf/homelessness-sans-abri/reports-rapports/data-shelter-2023-donnees-refuge-en.pdf

[bibr13-08445621251383062] Government of Canada . (2024). *Backgrounder: Action research on chronic homelessness (ARCH)*. https://www.canada.ca/en/housing-infrastructure-communities/news/2023/11/backgrounder-action-research-on-chronic-homelessness-arch.html

[bibr14-08445621251383062] Hartrick Doane G. Varcoe C. (2021). How to nurse: Relational inquiry in action (2nd ed.). Wolters Kluwer/Lippincott Williams & Wilkins.

[bibr15-08445621251383062] Homeless Prenatal Program . (2024). *Ending family poverty and homelessness: Family support*. https://www.homelessprenatal.org/family-support

[bibr16-08445621251383062] Hughes E. (1962). Good people and dirty work. Social Problems, 10(1), 3–11. 10.2307/799402

[bibr17-08445621251383062] International Council of Nurses . (2021). *The ICN code of ethics for nurses*. Geneva, Switzerland. https://admin.icn.ch/resources/publications-and-reports/icn-code-ethics-nurses

[bibr18-08445621251383062] Krahn J. Caine V. Chaw-Kant J. Singh A. E. (2018). Housing interventions for homeless, pregnant/parenting women with addictions: A systematic review. Journal of Social Distress and the Homeless, 27(1), 75–88. 10.1080/10530789.2018.1442186

[bibr19-08445621251383062] Lakhanpaul M. Svirydzenka N. (2024). Pregnant women experiencing homelessness struggle to access healthcare. BMJ, 387, q2126. 10.1136/bmj.q2126 39374973

[bibr20-08445621251383062] Lenzi M. Santinello M. Gaboardi M. Disperati F. Vieno A. Calcagnì A. Greenwood R. M. Rogowska A. M. Wolf J. R. Loubière S. Beijer U. Bernad R. Vargas-Moniz M. J. Ornelas J. Spinnewijn F. Shinn M. (2021). Factors associated with providers’ work engagement and burnout in homeless services: A cross-national study. American Journal of Community Psychology, 67(1–2), 220–236. 10.1002/ajcp.12470 33137234

[bibr21-08445621251383062] Paisi M. March-McDonald J. Burns L. Snelgrove-Clarke E. Withers L. Shawe J. (2020). Perceived barriers and facilitators to accessing and utilising sexual and reproductive healthcare for people who experience homelessness: A systematic review. BMJ Sexual & Reproductive Health, 47(3), 211–220. 10.1136/bmjsrh-2020-200799 33122258

[bibr22-08445621251383062] Purkey E. MacKenzie M. (2019). Experience of healthcare among the homeless and vulnerably housed: A qualitative study—opportunities for equity-oriented health care. International Journal for Equity in Health, 18, 101. 10.1186/s12939-019-1004-4 31262310 PMC6604349

[bibr23-08445621251383062] Riebe N. (2024, May 21). Fifteen years after the original strategy, Edmonton has a new plan for homelessness: Number of homeless people doubled since beginning of the COVID-19 pandemic. *CBC News*. https://www.cbc.ca/news/canada/edmonton/edmonton-homelessness-strategy-10-year-plan-1.7210726

[bibr24-08445621251383062] Roper J. Shapira J. (2000). Ethnography in nursing research. Sage Publishing.

[bibr25-08445621251383062] Schmidt C. N. Wingo E. E. Newmann S. J. Borne D. E. Shapiro B. J. Seidman D. L. (2023). Patient and provider perspectives on barriers and facilitators to reproductive healthcare access for women experiencing homelessness with substance use disorders in San Francisco. Women’s Health, 19, 10.1177/17455057231152374 PMC9947686 36939096

[bibr26-08445621251383062] Siegfried C. H. (1996). Pragmatism and feminism: Reweaving the social fabric. University of Chicago Press.

[bibr27-08445621251383062] Stanford Encyclopedia of Philosophy . (2025). Pragmatist feminism. In E. N., Zalta (Ed.), The Stanford Encyclopedia of Philosophy (Summer 2025 Edition). The Metaphysics Research Lab, Department of Philosophy, Stanford University. https://plato.stanford.edu/entries/femapproach-pragmatism/

[bibr28-08445621251383062] Stevens M. Clark M. Carlisle J. Brimblecombe N. MacGill M. (2024). Supporting meaningful implementation and evaluation of strengths-based approaches in adult social care: A theory of change for the three conversations. The British Journal of Social Work, 54(6), 2583–2602. 10.1093/bjsw/bcae055

[bibr29-08445621251383062] UNANIMA International . (2025). The hidden faces of homelessness: Global insights and pathways forward. United Nations. https://unanima-international.org/

[bibr30-08445621251383062] United Nations Development Programme . (2015). *The SDGs in action*. https://www.undp.org/sustainable-development-goals

[bibr31-08445621251383062] Women’s National Housing & Homelessness Network . (2025). *Women and girls’ homelessness in Canada*. https://womenshomelessness.ca/women-girls-homelessness-in-canada/

[bibr32-08445621251383062] World Health Assembly . (2025, May 27). The health of women, children, and adolescents: A key priority at the 78th World Health Assembly. https://pmnch.who.int/news-and-events/news/item/27-05-2025-the-health-of-women-children-and-adolescents-a-key-priority-at-the-78th-world-health-assembly

[bibr33-08445621251383062] World Population Review. (2025). Explore the world population through data. https://worldpopulationreview.com/

